# Reduced Segmentation of Lesions Is Comparable to Whole-Body Segmentation for Response Assessment by PSMA PET/CT: Initial Experience with the Keyhole Approach

**DOI:** 10.3390/biology11050660

**Published:** 2022-04-26

**Authors:** Philipp E. Hartrampf, Markus Krebs, Lea Peter, Marieke Heinrich, Julia Ruffing, Charis Kalogirou, Maximilian Weinke, Joachim Brumberg, Hubert Kübler, Andreas K. Buck, Rudolf A. Werner, Anna Katharina Seitz

**Affiliations:** 1 Department of Nuclear Medicine, University Hospital Würzburg, 97080 Würzburg, Germany; heinrich_m4@ukw.de (M.H.); joachim.brumberg@uniklinik-freiburg.de (J.B.); buck_a@ukw.de (A.K.B.); werner_r1@ukw.de (R.A.W.); 2 Department of Urology and Pediatric Urology, University Hospital Würzburg, 97080 Würzburg, Germany; krebs_m@ukw.de (M.K.); lea.peter@gmx.net (L.P.); julia.ruffing@web.de (J.R.); kalogirou_c@ukw.de (C.K.); weinke_m2@ukw.de (M.W.); kuebler_h@ukw.de (H.K.); seitz_a3@ukw.de (A.K.S.); 3 Comprehensive Cancer Center Mainfranken, University Hospital Würzburg, 97080 Würzburg, Germany; 4 Department of Nuclear Medicine, Faculty of Medicine, Medical Center-University of Freiburg, 79106 Freiburg, Germany

**Keywords:** PET/CT, PSMA-TV, SUV, prostate cancer, taxane, radioligand therapy

## Abstract

**Simple Summary:**

The calculation of PSMA-positive tumor volume (PSMA-TV) of the whole body from PSMA PET scans for response evaluation remains a time-consuming procedure. We hypothesized that it may be possible to quantify changes in PSMA-TV by considering only a limited number of representative tumor lesions. Changes in the whole-body PSMA-TV of 65 patients were comparable to the changes in PSMA-TV after including only the ten largest lesions. Moreover, changes in PSMA-TV correlated well with changes in PSA levels, as did the changes in PSMA-TV with the reduced number of lesions. We conclude that a response assessment using PSMA-TV with a reduced number of lesions is feasible and could lead to a simplified process for evaluating PSMA PET/CT.

**Abstract:**

(1) Background: Prostate-specific membrane antigen (PSMA) positron emission tomography (PET)-derived parameters, such as the commonly used standardized uptake value (SUV) and PSMA-positive tumor volume (PSMA-TV), have been proposed for response assessment in metastatic prostate cancer (PCa) patients. However, the calculation of whole-body PSMA-TV remains a time-consuming procedure. We hypothesized that it may be possible to quantify changes in PSMA-TV by considering only a limited number of representative lesions. (2) Methods: Sixty-five patients classified into different disease stages were assessed by PSMA PET/CT for staging and restaging after therapy. Whole-body PSMA-TV and whole-body SUV_max_ were calculated. We then repeated this calculation only including the five or ten hottest or largest lesions. The corresponding serum levels of prostate-specific antigen (PSA) were also determined. The derived delta between baseline and follow-up values provided the following parameters: ΔSUV_maxall_, ΔSUV_max10_, ΔSUV_max5_, ΔPSMA-TV_all_, ΔPSMA-TV_10_, ΔPSMA-TV_5_, ΔPSA. Finally, we compared the findings from our whole-body segmentation with the results from our keyhole approach (focusing on a limited number of lesions) and correlated all values with the biochemical response (ΔPSA). (3) Results: Among patients with metastatic hormone-sensitive PCa (mHSPC), none showed a relevant deviation for ΔSUV_max10_/ΔSUV_max5_ or ΔPSMA-TV_10_/ΔPSMA-TV_5_ compared to ΔSUV_maxall_ and ΔPSMA-TV_all_. For patients treated with taxanes, up to 6/21 (28.6%) showed clinically relevant deviations between ΔSUV_maxall_ and ΔSUV_max10_ or ΔSUV_max5_, but only up to 2/21 (9.5%) patients showed clinically relevant deviations between ΔPSMA-TV_all_ and ΔPSMA-TV_10_ or ΔPSMA-TV_5_. For patients treated with radioligand therapy (RLT), up to 5/28 (17.9%) showed clinically relevant deviations between ΔSUV_maxall_ and ΔSUV_max10_ or ΔSUV_max5_, but only 1/28 (3.6%) patients showed clinically relevant deviations between ΔPSMA-TV_all_ and ΔPSMA-TV_10_ or ΔPSMA-TV_5_. The highest correlations with ΔPSA were found for ΔPSMA-TV_all_ (r ≥ 0.59, *p* ≤ 0.01), followed by ΔPSMA-TV_10_ (r ≥ 0.57, *p* ≤ 0.01) and ΔPSMA-TV_5_ (r ≥ 0.53, *p* ≤ 0.02) in all cohorts. ΔPSA only correlated with ΔSUV_maxall_ (r = 0.60, *p* = 0.02) and with ΔSUV_max10_ (r = 0.53, *p* = 0.03) in the mHSPC cohort, as well as with ΔSUV_maxall_ (r = 0.51, *p* = 0.01) in the RLT cohort. (4) Conclusion: Response assessment using PSMA-TV with a reduced number of lesions is feasible, and may allow for a simplified evaluation process for PSMA PET/CT.

## 1. Introduction

More than ever, the discovery and development of new treatment strategies for metastatic prostate cancer (PCa) is an emerging focus in uro-oncology. For all treatment strategies, it is critical to determine drug efficacy and to estimate the survival benefit for patients by distinguishing between responders and non-responders. The mainstay of response assessment in metastatic PCa are the Prostate Cancer Clinical Trials Working Group (PCWG3) criteria [1], which include clinical and laboratory parameters as well as conventional imaging techniques.

Conventional imaging techniques such as computed tomography (CT) show some weaknesses in therapy response evaluation. For example, blastic bone lesions are not measurable using the established Response Evaluation Criteria in Solid Tumors 1.1 (RECIST 1.1) [2]. By adding metabolic information to conventional imaging, prostate-specific membrane antigen (PSMA) PET/CT seems to be superior to CT, which has been corroborated for detecting recurrence [3,4] and assessing therapy response [5]. However, identifying responders on PSMA PET/CT also poses challenges for clinicians. To address the need for reporting standards, expert consensus statements were published in 2021 to initiate the development of guidelines for molecular imaging with PSMA PET/CT [6].

For the quantification of PSMA PET/CT, standardized uptake values (SUVs) and PET-positive tumor volume (TV)—also referred to as PSMA tumor volume (PSMA-TV)—are commonly used [7]. In this regard, several studies have demonstrated that post-therapeutic changes in PSMA-TV correlate with biochemical responses (BRs) [7,8], particularly for osseous lesions. Of note, for skeletal involvement, PSMA-TV derived from PSMA PET/CT outperformed CT for correlation with BR, thereby indicating a tight link between molecular-imaging-based TV and response to prostate-cancer-specific treatment [3]. In addition, changes in PSMA-TV and SUV were also associated with PSA response in metastatic PCa patients undergoing various systemic therapies (radium-223, taxane-based chemotherapy, abiraterone, enzalutamide) [9].

A weakness of whole-body PSMA-TV acquisition is that it is a time-consuming process, despite the use of algorithms for the semi-automatic quantification of tumor volume in PSMA PET/CT [10] and the use of additional neural networks [11]. To overcome this obstacle, we hypothesized that it may not be necessary to calculate the whole-body PSMA-TV and SUV_max_ to provide a reliable read-out of their changes.

In the present investigation, we calculated the entire whole-body PSMA-TV and SUV_max_ from the PSMA PET/CTs of 65 patients. We then reduced the number of measured lesions to include those with the highest SUV_max_ and the largest volume (“keyhole approach”). Using both approaches, we investigated and compared the changes induced by the therapy. Finally, the therapy-induced changes in PSMA-TV and SUV_max_ were correlated with the delta of serum PSA levels. Again, the whole-body approach and the “keyhole” approach were also compared.

## 2. Materials and Methods

### 2.1. Study Cohort

All patients who received [^68^Ga] Ga-PSMA I&T PET/CT (PSMA PET/CT) for staging and restaging at our hospital between July 2014 and December 2018 were screened. Inclusion criteria were at least one PSMA PET/CT in a three-month period before therapy (“baseline”) and another scan in a four-month period after completion/termination of therapy or after one cycle of radioligand therapy (“follow-up”). At the respective time points, the corresponding serum levels of prostate-specific antigen (PSA) were determined. Detailed characteristics of the study cohort (*n* = 65) are shown in Table 1.

All findings, data acquisition and processing in this study comply with the ethical standards stipulated in the latest Declaration of Helsinki, as well as with the statutes of the Ethics Committee of the University of Würzburg concerning anonymized retrospective medical studies. Ethical review and approval were waived for this study by the local Ethics Committee due to the retrospective nature of the study (waiver no. 20, 191, 106 02).

### 2.2. PSMA PET/CT Imaging Protocol

The PET/CT images were obtained with [^68^Ga] Ga-PSMA I&T. The imaging protocol and in-house labelling were performed as described elsewhere [12]. Briefly, patients underwent PSMA PET/CT from the skull base to the mid-thigh using a Biograph mCT scanner (Siemens Medical Solutions, Erlangen, Germany). The PET/CT included a diagnostic CT scan in the portal venous phase.

### 2.3. PSMA PET/CT Analysis

PSMA PET/CT images were analyzed using the Beth Israel plugin for FIJI (ImageJ) [13], a freely available shareware from the Beth Israel Deaconess Medical Center (Boston, MA, USA), Division of Nuclear Medicine and Molecular Imaging. We performed the semi-automatic analysis with FIJI using the automatic segmentation function, as described by the developers and in [12]. In brief, a 3 cm spherical region of interest (ROI) in the liver was set as the threshold based on PERCIST and PROMISE criteria (threshold: 1.5 × liver mean + 2 × standard deviation) [14,15]. In patients with known liver metastases, the threshold was based on an ROI with a diameter of 1 cm in the descending thoracic aorta extending over a *z*-axis of 2 cm (threshold: 2 × aortic mean + 2 × standard deviation). After automatic analysis, lesion-based visual inspection was performed by at least two investigators (P.E.H., M.H., L.P.) and the segmentations were manually corrected. For each lesion, maximum standardized uptake value (SUV_max_) and PSMA-positive tumor volume were determined. In addition, the hottest lesion (highest SUV_max_ of all lesions) and the number of measurable lesions were determined for each patient. The sum of all lesions yielded the whole-body SUV_max_ (SUV_maxall_) and the whole-body PSMA-positive tumor volume (PSMA-TV_all_) for each patient.

### 2.4. Response Assessment

Relative changes in the summed SUV_maxall_ (ΔSUV_maxall_) and the summed PSMA-TV_all_ (ΔPSMA-TV_all_) as well as changes in serum PSA levels (ΔPSA) were calculated by comparing the values at follow-up with the values at baseline (rel. ΔX(%) = X_follow-up_/X_baseline_ × 100 − 100). We then reduced the number of lesions. For SUV_max_, we used the ten and the five hottest lesions (SUV_max10_, SUV_max5_) and for PSMA-TV, we used the ten and the five largest lesions (PSMA-TV_10_, PSMA-TV_5_). For these parameters, the differences between the baseline and follow-up values were calculated and named accordingly (ΔSUV_max10_, ΔSUV_max5_, ΔPSMA-TV_10_, ΔPSMA-TV_5_). Post-treatment changes were interpreted according to the PET Response Criteria in Solid Tumors (PERCIST) 1.0 [14] and the consensus guidelines [6]. Changes in the summed SUV_max_ or the summed PSMA-TV ≥ +30% were considered progressive disease (PD) and ≤ +30% were considered responders. The latter were divided into partial response (PR; a decrease of ≥30%) and stable disease (SD; between −30% and +30%).

Finally, we compared the results obtained after considering the reduced number of lesions with those obtained after considering all lesions. We regarded a discrepancy between PD and SD/PR as clinically relevant as this would lead to a change in a patient’s treatment. Accordingly, discrepancies between PR and SD were regarded as clinically non-relevant.

### 2.5. Statistical Analysis

We performed statistical analyses with GraphPad Prism version 9.3.0 for Windows (GraphPad Software, San Diego, CA, USA) and applied Shapiro–Wilk tests for normal distribution. Due to the non-normal distribution of the data, we used Spearman’s rank correlation coefficient for correlation analysis. A *p*-value less than 0.05 was considered statistically significant.

## 3. Results

### 3.1. Metastatic Hormone-Sensitive Prostate Cancer

**Figure 1:**
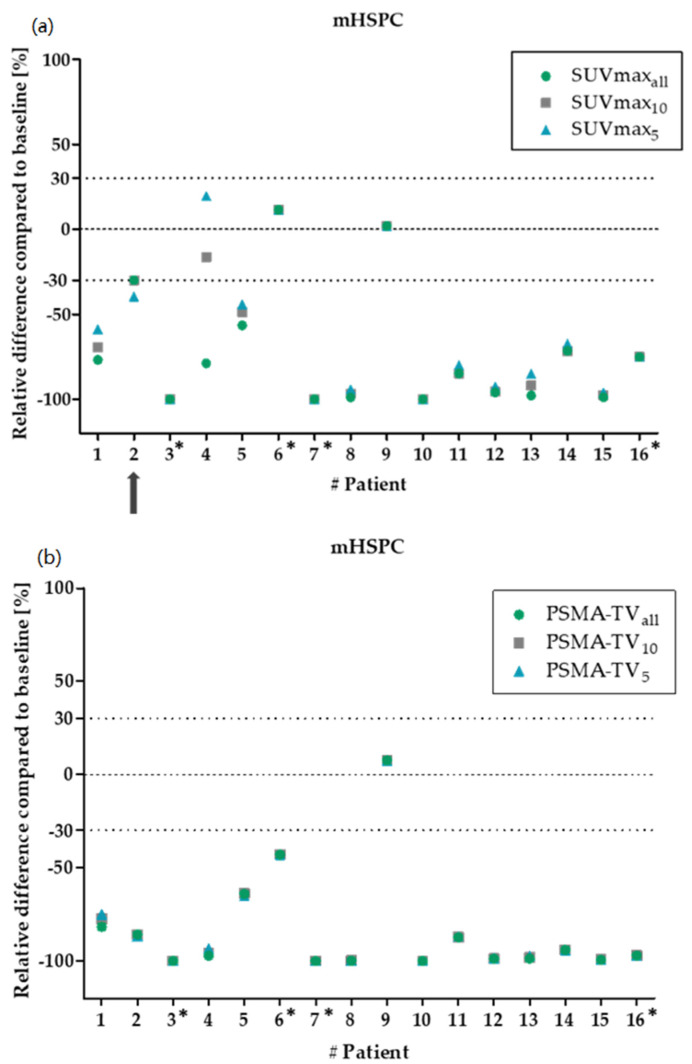
Relative changes between baseline and follow-up for SUV_max_ (**a**) and PSMA-TV (**b**) in patients with metastatic hormone-sensitive prostate cancer (mHSPC). The green dots show the changes for all segmented lesions. The grey squares/blue triangles show the ten/five hottest lesions for SUV_max_ and the ten/five largest lesions for PSMA-TV. The dotted lines mark the borders, which are considered as clinically relevant (±30%). No clinically relevant deviations were found between the segmentation of all lesions and the reduced lesions. The black arrow indicates a clinically non-relevant deviation in one patient for SUV_max_. The asterisks mark the patients with less than five lesions.

Initially, patients with metastatic hormone-sensitive PCa (mHSPC) were used as a training cohort because treatment response can be expected in therapy-naïve patients. None of the 16 patients suffering from mHSPC revealed a clinically relevant deviation in ΔSUV_max_. Neither ΔSUV_max10_ nor ΔSUV_max5_ showed different results compared to ΔSUV_maxall_. Only one patient showed a clinically non-relevant difference between PR and SD (Figure 1a). For ΔPSMA-TV, none of the 16 patients showed a relevant difference from ΔPSMA-TV_all_, neither for ΔPSMA-TV_10_ nor for ΔPSMA-TV_5_ (Figure 1b).

### 3.2. Metastatic Castration-Resistant Prostate Cancer–Taxane-Based Therapy

**Figure 2:**
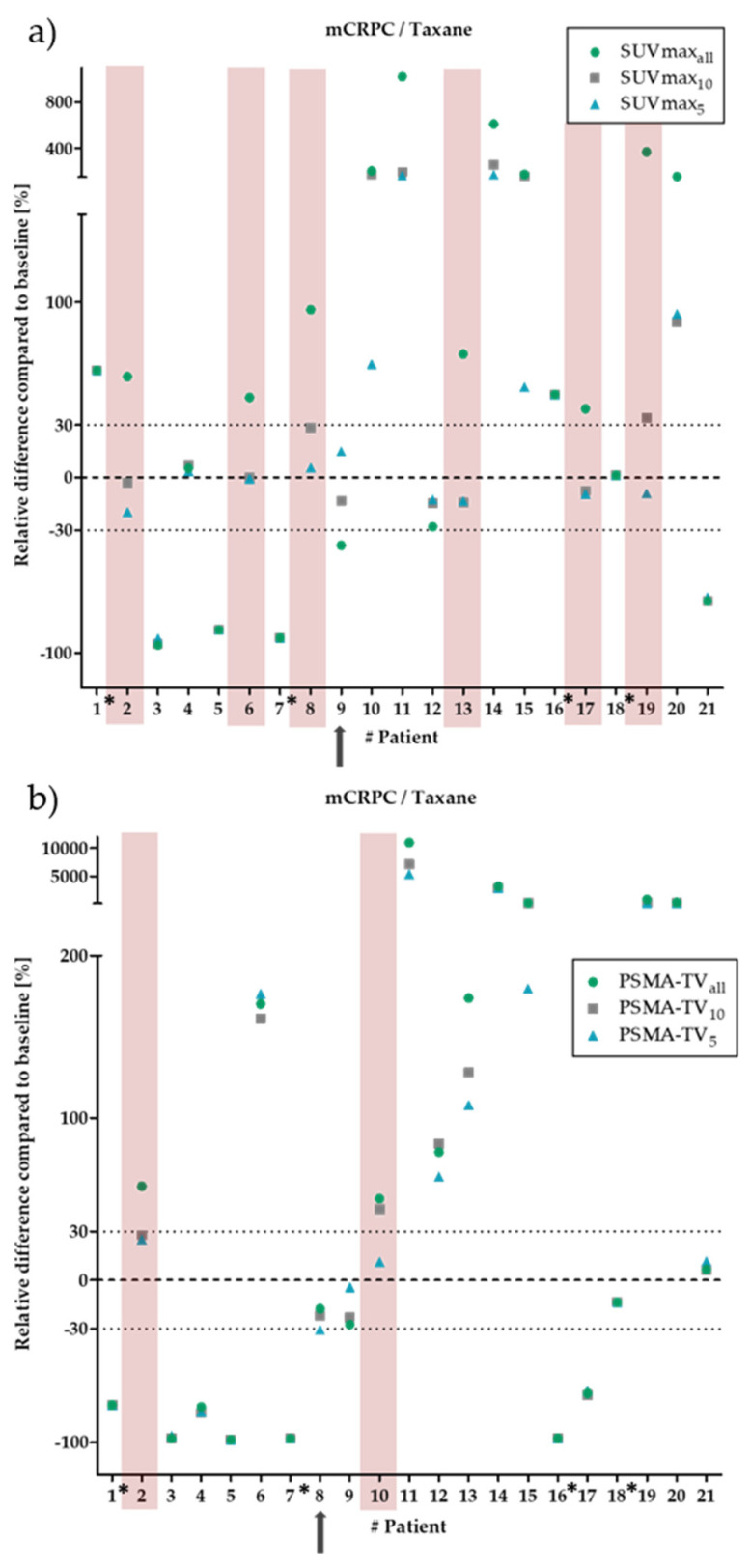
Relative changes between baseline and follow-up for SUV_max_ (**a**) and PSMA-TV (**b**) in patients with metastatic castration-resistant prostate cancer (mCRPC) undergoing taxane therapy. The green dots show the changes for all segmented lesions. The grey squares/blue triangles show the ten/five hottest lesions for SUV_max_ and the ten/five largest lesions for PSMA-TV. The dotted lines mark the borders, which are considered clinically relevant (±30%). The red bars mark patients with a clinically relevant deviation. For SUV_max_, 6 of the 21 patients showed a clinically relevant deviation. For PSMA-TV, 19 of the 21 patients showed no clinically relevant deviation. The black arrows indicate a clinically non-relevant deviation in one patient. The asterisks mark the patients with less than five lesions.

We then attempted to validate the keyhole approach in cohorts with higher tumor burden and castration-resistant PCa. For patients undergoing taxane-based therapy, the ΔSUV_max_ showed a clinically relevant deviation in 6 of the 21 patients. In all these differing cases, ΔSUV_maxall_ marked PD, while ΔSUV_max5_ resulted in SD classification. For ΔSUV_max10_, five of these six patients were classified with SD. In addition, a non-clinically relevant difference between response and stable disease was observed in one patient (Figure 2a).

For ΔPSMA-TV, 19 of the 21 patients showed no relevant deviation from ΔPSMA-TV_all_ for either ΔPSMA-TV_10_ or ΔPSMA-TV_5_. In the other patients, there was a clinically relevant deviation between PD for ΔPSMA-TV_all_ and SD for ΔPSMA-TV_5_ (two patients) and ΔPSMA-TV_10_ (one patient). One patient had a clinically non-relevant deviation in which ΔPSMA-TV_5_ showed a PR, while ΔPSMA-TV_10_ and ΔPSMA-TV_all_ showed SD (Figure 2b).

### 3.3. Metastatic Castration-Resistant Prostate Cancer–RLT

**Figure 3:**
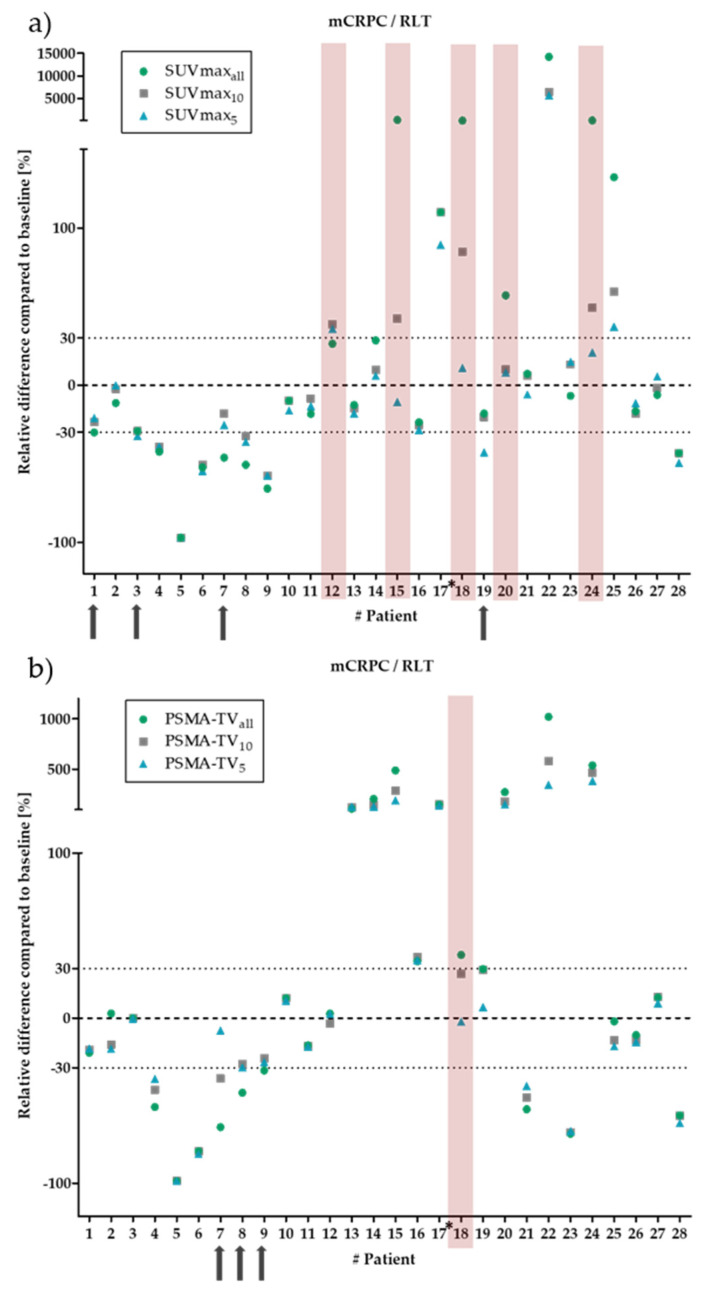
Relative changes between baseline and follow-up for SUV_max_ (**a**) and PSMA-TV (**b**) in patients with metastatic castration-resistant prostate cancer (mCRPC) undergoing radioligand therapy (RLT). The green dots show the changes for all segmented lesions. The grey squares/blue triangles show the ten/five hottest lesions for SUV_max_ and the ten/five largest lesions for PSMA-TV. The dotted lines mark the borders, which are considered as clinically relevant (±30%). The red bars mark patients with a clinically relevant deviation. For SUV_max_, 5 of the 28 patients showed a clinically relevant deviation. For PSMA-TV, only 1 of the 28 patients showed a relevant deviation. The black arrows indicate clinically non-relevant deviations in four patients for SUV_max_ and three patients for PSMA-TV. The asterisks mark the patients with less than five lesions.

For patients undergoing RLT, the ΔSUV_max_ showed a clinically relevant deviation in 5 of the 28 patients. While ΔSUV_maxall_ values marked PD in four patients, ΔSUV_max5_ resulted in SD classification for all four patients and ΔSUV_max10_ showed SD in one of these four patients. The other three patients showed PD at ΔSUV_max10_, in agreement with ΔSUV_maxall_. One patient showed SD at ΔSUV_maxall_ but PD at ΔSUV_max10_ and ΔSUV_max5_. In addition, a clinically non-relevant difference between PR and SD was observed in four patients (Figure 3).

For ΔPSMA-TV, only 1 of the 28 patients showed a relevant deviation, with a difference between progression in ΔPSMA-TV_all_ and stable disease in ΔPSMA-TV_10_ and ΔPSMA-TV_5_. Four patients showed a clinically non-relevant deviation with differences between PR and SD (Figure 3).

### 3.4. Correlations of Changes in PSMA-TV and SUV_max_ with Changes in PSA Values

The results of correlation analyses for ΔPSA and ΔPSMA-TV or ΔSUV_max_ are summarized in Table 2.

For ΔPSMA-TV, the highest correlation coefficients were found for ΔPSMA-TV_all_, followed closely by ΔPSMA-TV_10_ in all cohorts. ΔPSMA-TV_5_ had lower correlation coefficients, but these were still strong and significant.

For ΔSUV_max_, significant correlations were found only in the mHSPC and RLT cohorts. The ΔPSA correlated with ΔSUV_maxall_ as well as with ΔSUV_max10_ in the mHSPC cohort. In the RLT cohort, ΔPSA only correlated significantly with ΔSUV_maxall_.

Regarding the change in the hottest lesions only, there was a significant correlation with changes in PSA levels in the mHSPC cohort, whereas the other cohorts did not show significant correlations.

## 4. Discussion

In this study, we developed a simplified evaluation procedure—the so-called keyhole approach—and investigated whether this approach still meets the clinical requirements of response assessment. We demonstrated that ΔPSMA-TV correlated significantly with ΔPSA. Focusing on the ten largest lesions had no clinically meaningful impact on response assessment (SD, PR, PD) for ΔPSMA-TV or correlations with ΔPSA. In contrast, the informative value for the assessment of the response seemed rather limited for ΔSUV_max_.

In the subgroup of patients with mHSPC, changes in the reduced number of lesions showed the same trend as whole-body segmentation. As this accordance might be a result of a small number of lesions, we also counted the lesions of each patient. The median number of lesions in this cohort was 11, with a range between 1 and 89. Eight patients had more than ten lesions, whereas the remaining eight patients had between one and nine lesions. Correlation with ΔPSA was best for ΔPSMA-TV_all_, with almost no difference from ΔPSMA-TV_10_ and only variances for ΔPSMA-TV_5_. The correlation of ΔPSA with ΔSUV_maxall_ was weak, and this correlation was even less pronounced for ΔSUV_max10_ and ΔSUV_max5_. Nonetheless, we believe that reducing the number of lesions is feasible for these patients and shows comparable results to the whole-body approach assessing the entire tumor burden.

In contrast to the mHSPC cohort, our keyhole approach did not provide convincing results for the ΔSUV_max_ in patients with mCRPC. We found clinically relevant differences in 6/21 patients in the taxane group and in 5/28 patients in the RLT group. As such, this approach should not be implemented in clinical practice, and thus we cannot recommend reducing the number of lesions for obtaining ΔSUV_max_. One explanation for this phenomenon could be that novel lesions may skew the results, especially when the original number of lesions is low and when novel lesions appear to be very intense. In general, a low initial number of lesions is likely to result in a larger deviation, as the appearance of new sites of disease may have a greater impact in the context of providing SUV_max_.

For ΔPSMA-TV, however, focusing on the ten or five largest lesions worked well and the best results were achieved when including ten metastases.

Correlations of ΔPSA were markedly higher for ΔPSMA-TV compared to ΔSUV_max_ in both subgroups of mCRPC patients. A substantial association with biochemical responses was recorded when focusing on the ten largest lesions, whereas focusing on the five largest lesions resulted in rather weak but still significant correlations. Correlation coefficients were slightly higher in the RLT cohort compared to the taxane cohort. This may be partially explained by the use of a more standardized restaging protocol for the RLT group, in which restaging was performed in all patients after the first cycle. In contrast, restaging in the taxane group was performed after completion of therapy but not at well-defined time points, as conducted for patients scheduled for RLT. In this regard, the total lesion number had no impact because the number of lesions in the taxane cohort was comparable to the mHSPC cohort, whereas it was significantly higher in the RLT group.

A future goal should be to develop a response assessment system for PSMA PET/CT, similar to RECIST for CT. In this context, the clinical significance of only a few new PSMA-positive lesions is unclear and not well studied. The current consensus is that a new lesion without a relevant change in whole-body tumor volume (defined as increase of 30%) on PSMA PET/CT should not be considered as progressive disease [6]. Based on our findings, we recommend considering the PSMA-positive TV for response assessment instead of SUV_max_. Our assumption is that the tumor volume is less susceptible to changes caused by a small number of lesions.

Detecting the hottest and largest lesions in our approach was easy, as we had a whole-body segmentation containing all lesions and could select lesions from a ranking list. In clinical practice, readers usually do not have the option to select from such a ranking list. Instead, they must identify suitable lesions based on the scan. This presents another challenge for the future, including how to identify appropriate lesions for response assessment, in particular whether to use only the hottest lesion (as proposed for FDG in PERCIST) [14] or target lesions (according to RECIST) [2]. Therefore, after using the largest and hottest lesions in this study, the next step may be to evaluate the definitions of specific target lesions.

The retrospective nature of the study and the lack of fully standardized imaging protocols for the different cohorts are limitations of the study. In addition, we used a threshold based on the SUV_mean_ of the liver. As a result, some lesions within this threshold may have been missed. On the other hand, segmented lesions are more likely to mark PCa lesions. In addition, we correlated PET response to BR, as serum PSA levels should be assessed in accordance with the recommendations for treatment response in advanced PCa [1]. However, changes in PSA levels during systemic treatment should be carefully interpreted [16] and PSA levels alone may not be sufficiently reliable for monitoring disease activity, especially in mCRPC patients. Conversely, mCRPC patients are more likely to develop PSMA-negative metastases after various therapies due to increasing tumor heterogeneity. These PSMA-negative metastases are missed by PSMA-targeted imaging and the changes may contrast with PSA levels.

## 5. Conclusions

When assessing changes in PSMA-TV, it is feasible to focus on a reduced number of lesions. Notably, the correlation with PSA response was comparable to changes of a whole-body PSMA-TV approach that covers the entire tumor burden. These results could simplify the evaluation process when using PSMA PET/CT to evaluate PSMA-positive TV.

## Figures and Tables

**Table 1:** Patient characteristics.

	All Patients (*n* = 65)	mHSPC (*n* = 16)	Taxane Group (*n* = 21)	PSMA RLT Group (*n* = 28)
Age (years)	71 (54–93)	66 (54–83)	72 (55–93)	72 (54–90)
Gleason score	8 (6–10)	8 (7–9)	9 (6–10)	9 (7–10)
PSA (ng/mL)	60.5 (0.54–3130)	89.5 (9.80–1239)	17.8 (0.54–800)	166 (5.74–3130)
Sites of disease	*n* (patients)	*n* (patients)	*n* (patients)	*n* (patients)
Prostate/local	25	16	4	5
Lymph node	49	13	18	18
Bone	56	13	16	27
Liver	8	0	4	4
Lung	6	3	2	1
Prior treatments	*n* (patients)	*n* (patients)	*n* (patients)	*n* (patients)
Prostatectomy	26	0	12	14
Radiotherapy to prostate/prostate bed	6	0	3	3
ADT	64 *	16	21	27 *
Abiraterone	36	7	8	21
Enzalutamide	17	0	0	17
Docetaxel	41	9	15	17
Cabazitaxel	13	0	7	6
[^223^Ra] Dichloride	6	0	2	4
PSMA RLT	28	0	0	28
Number of segmented baseline lesions	13 (1–144)	11 (1–89)	10 (1–63)	29 (4–144)

mHSPC = metastatic hormone-sensitive prostate cancer, PSA = prostate-specific antigen, ADT = androgen deprivation therapy, PSMA RLT = prostate-specific membrane antigen radioligand therapy, * one patient had orchiectomy.

**Table 2:** Spearman rank correlation coefficients for ΔSUV_max_/ΔPSMA-TV with therapy-induced PSA changes (ΔPSA).

	ΔPSA (%) vs. ΔPSMA-TV (%)		ΔPSA (%) vs. ΔSUV_max_ (%)		ΔPSA (%) vs. ΔSUV_max_ Hottest Lesion (%)	
	Spearman r	*p*-Value	Spearman r	*p*-Value	Spearman r	*p*-Value
**mHSPC**						
total	0.63	0.01	0.60	0.02	0.53	0.04
ten largest	0.63	0.01				
five largest	0.62	0.01				
ten hottest			0.53	0.03		
five hottest			0.48	0.06		
**Taxane-based** **therapy**						
total	0.59	0.01	0.47	0.05	0.26	0.27
ten largest	0.57	0.01				
five largest	0.53	0.02				
ten hottest			0.21	0.40		
five hottest			0.14	0.58		
**Radioligand therapy**						
total	0.62	<0.01	0.51	**0.01**	0.29	0.14
ten largest	0.60	<0.01				
five largest	0.55	<0.01				
ten hottest			0.37	0.06		
five hottest			0.22	0.27		

## Data Availability

The main data presented in this study are available in the article. Detailed information about the image analysis or the overall survival of the subjects presented in this study are available on reasonable request from the corresponding author.
